# A Brief Review of MoO_3_ and MoO_3_-Based Materials and Recent Technological Applications in Gas Sensors, Lithium-Ion Batteries, Adsorption, and Photocatalysis

**DOI:** 10.3390/ma16247657

**Published:** 2023-12-15

**Authors:** Mário Gomes da Silva Júnior, Luis Carlos Costa Arzuza, Herbet Bezerra Sales, Rosiane Maria da Costa Farias, Gelmires de Araújo Neves, Hélio de Lucena Lira, Romualdo Rodrigues Menezes

**Affiliations:** Laboratory of Materials Technology (LTM), Department of Materials Engineering, Federal University of Campina Grande (UFCG), Av. Aprígio Veloso 882, Campina Grande 58429-900, PB, Brazil; luisarzuza179@gmail.com (L.C.C.A.); herbet_bezerra@hotmail.com (H.B.S.); rosiane.farias@ufcg.edu.br (R.M.d.C.F.); gelmires.neves@ufcg.edu.br (G.d.A.N.); helio.lucena@professor.ufcg.edu.br (H.d.L.L.)

**Keywords:** MoO_3_-based materials, gas sensors, lithium-ion batteries, adsorption, photocatalysis

## Abstract

Molybdenum trioxide is an abundant natural, low-cost, and environmentally friendly material that has gained considerable attention from many researchers in a variety of high-impact applications. It is an attractive inorganic oxide that has been widely studied because of its layered structure, which results in intercalation ability through tetrahedral/octahedral holes and extension channels and leads to superior charge transfer. Shape-related properties such as high specific capacities, the presence of exposed active sites on the oxygen-rich structure, and its natural tendency to oxygen vacancy that leads to a high ionic conductivity are also attractive to technological applications. Due to its chemistry with multiple valence states, high thermal and chemical stability, high reduction potential, and electrochemical activity, many studies have focused on the development of molybdenum oxide-based systems in the last few years. Thus, this article aims to briefly review the latest advances in technological applications of MoO_3_ and MoO_3_-based materials in gas sensors, lithium-ion batteries, and water pollution treatment using adsorption and photocatalysis techniques, presenting the most relevant and new information on heterostructures, metal doping, and non-stoichiometric MoO_3−x_.

## 1. Introduction

**Figure 1:**
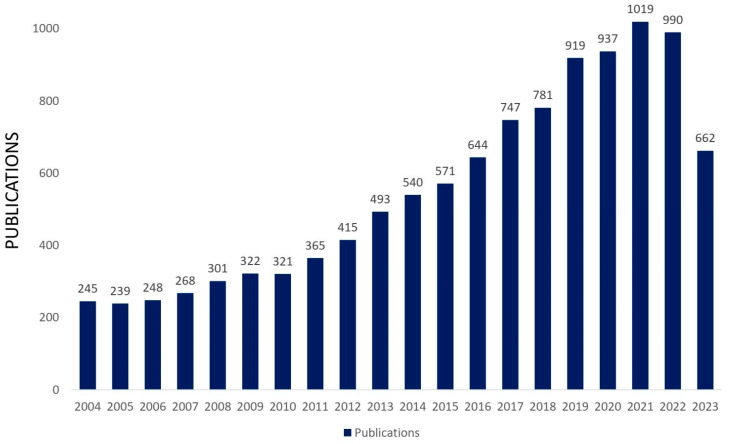
Published scientific papers directly related to MoO_3_ and MoO_3_-based systems in the last 20 years (Web of Science search with MoO_3_ keyword, 2023 ongoing, Access on 9 October 2023).

Molybdenum oxides are versatile compounds that have attracted the attention of many researchers worldwide since they are one of the most adaptable and functional optical and electronic oxides [1]. Among these oxides, MoO_3_ is an abundant natural and sustainable material that has gained considerable attention due to its chemistry related to multivalent Mo, high thermal and chemical stability, and redox chemistry resulting from its high reduction potential and electrochemical activity [2,3,4,5]. Due to its large band gap and optical and electrical properties, MoO_3_ is considered a potential and appropriate material for various technological applications in the fields of photocatalysis, adsorption, gas sensing, battery electrodes as anodic and cathodic materials, recording material, electrochromic and photochromic materials, organic solar cells, and organic light-emitting diodes as a buffer layer and as a catalyst for the electrochemical reduction in several ions [6,7,8]. Interestingly, MoO_3_ and MoO_3−x_ oxides have been used as hole injection/extraction interlayers in organic solar cells in order to modify the anode contact due to their high work function and their ability to exchange charges with several semiconductors, which requires the energy-level alignment of the materials. As a result, there is a reduction in the barrier for hole extraction from the organic photoactive layer to the metallic anode through the formation of a large interfacial dipole [9,10,11]. MoO_3_-based materials are also widely used in various industries as display devices and smart windows and are an excellent anti-friction and anti-wear agent as a lubricant additive [2,12,13]. Its absorption band is close to the maximum of human eye sensitivity. Furthermore, it also exhibits a better electrochromic response than other oxides when colored, owing to more intense and regular light absorption [14]. This high applicability is confirmed by verifying the many studies carried out in different technological segments like nanotechnology, energy fuels, electrochemistry, environmental science, optics, etc. An increasing trend in the last 20 years in papers directly related to MoO_3_ and MoO_3_-based systems has been verified (Figure 1).

**Figure 2:**
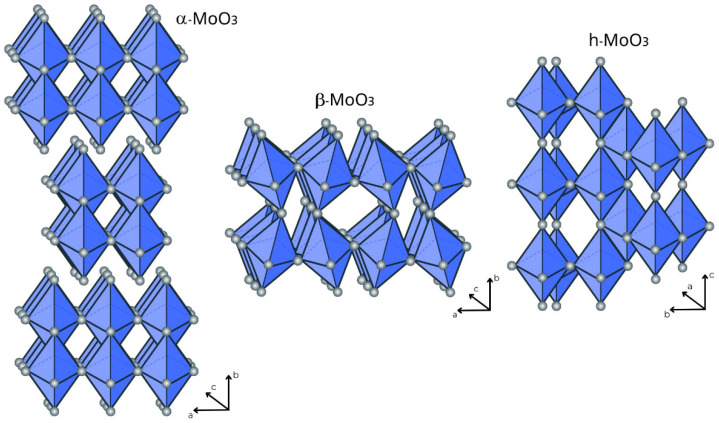
MoO_3_ crystalline structure illustrations.

Molybdenum has oxidation states ranging from +2 to +6. However, molybdenum oxides are primarily found in two forms: Mo (IV) oxide and Mo (VI) oxide [15]. Being the most usual, MoO_3_ is characterized by three different crystalline polymorphs: thermodynamically stable orthorhombic (α-MoO_3_), metastable monoclinic (β-MoO_3_), and low-temperature metastable hexagonal (h-MoO_3_) [2,15,16,17,18]. The different MoO_3_ crystalline structures are illustrated in Figure 2. In particular, α-MoO_3_ is an n-type indirect semiconductor with a wide band gap (2.8–3.6 eV) depending on its oxygen vacancy content [15,18,19]. It has high ionic conductivity due to its natural tendency toward oxygen vacancy formation and high stability in the air [5,7]. Moreover, one of the distinguishing characteristics of α-MoO_3_ is its intrinsic layered nature, which allows large quantities of positive ions to be accommodated in its tetrahedral and octahedral holes and extension channels, allowing bandgap manipulations. Furthermore, the layered structure also leads to superior charge transfer [5,20].

**Figure 3:**
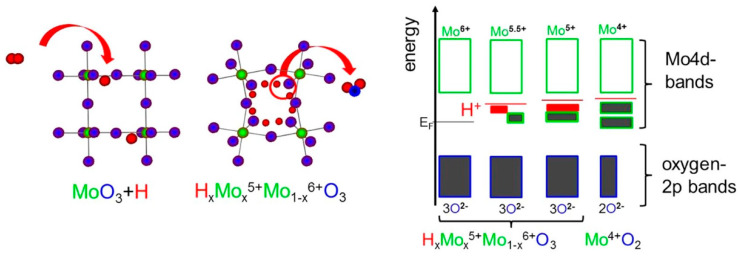
**Right** panel: Crystal structure of MoO_3_ with H+ intercalation and hydrogen molybdenum bronze. **Left** panel: Electronic band structure manipulation by H+ intercalation. Figure reproduced with permission of [24] Copyright 2017, Nature Publishing Group.

The properties and oxidation states of these phases change with temperature and energy supply, making the fabrication of various molybdenum oxide-based materials possible [21]. Besides MoO_3_, the crystallographic phases of molybdenum oxides include MoO_2_, Mo_8_O_23_, and Mo_4_O_11_ [21,22]. To engineer and control the electronic states of molybdenum oxides, oxidation state variations obtained from oxygen vacancy formation and dopant placement are used to enable crystal structure and morphology manipulation [1]. In particular, oxygen vacancy insertion is one of the best ways to control the doping levels in molybdenum oxides. This is because different amounts of oxygen vacancies can be made with high precision, controlling the change from semiconducting to metallic properties. Specifically, the electronic and optical properties of the MoO_3_ semiconducting oxide are significantly changed by the oxygen vacancies insertion. Regarding the non-stoichiometric MoO_3−x_, when 0 < x < 0.125, this oxide exhibits semiconducting properties, while 0.125 < x < 1 exhibits quasimetallic properties, and for x = 1, it creates semiconducting MoO_2_ with a reduced bandgap or semimetallic [1]. To comprehend the reactions that take place on the MoO_3−x_ surfaces, it is essential to understand the stability, shape, and replenishment techniques of vacancy creation [1]. One efficient technique to create a gap state in MoO_3_ is to anneal it in reducing gas, which removes the oxygen ions from its rigid metal-oxygen network [23]. The creation and control of oxygen vacancies can also be achieved by H+ intercalating into molybdenum oxide, forming the hydrogen molybdenum bronzes H_x_Mox^5+^Mo_1_−_x_^6+^O_3_ [24]. Hydrogenous intercalation does not considerably modify the crystalline structure, but there are small changes in volume, lattice distortion, and ordering of H (right panel, Figure 3). Furthermore, due to the proximity between hydrogen and oxygen, water is formed, changing the metal/oxygen ratio and, consequently, the electronic structure. The left panel of Figure 3 shows schematically the effect on the band structure of H+ intercaled into MoO_3_.

The semiconducting MoO_2_ has a monoclinic lattice system and can be made by removing the stoichiometric oxygen from MoO_3_ in a C or H_2_ reduction atmosphere. Because of its increased free electrons associated with oxygen vacancy richness, MoO_2_ has stronger metallic properties than semiconducting properties, with a significantly reduced bandgap [1]. The reaction that reduces MoO_3_ to MoO_2_ can be explained in two ways. In the first one, the MoO_3_ reduction initiates with the creation of oxygen vacancies arranged as shear planes as a consequence of the crystallographic shear that aggregates into discs. As the concentration of the shear planes increases, they order and form the Magnelli phases (MonO_3n−1_) as an intermediate product, an orthorhombic Mo_4_O_11_ [25,26,27]. At higher temperatures, the structure is then converted to MoO_2_ due to the formation of a critical oxygen vacancy concentration [25]. Thereby, the rate-controlling steps of MoO_3_ to Mo_4_O_11_ and Mo_4_O_11_ to MoO_2_ are the interfacial chemical reactions that obey the nucleation and growth models and the temperature-dependent processes that obey the diffusion models, respectively [27]. The second explanation is that the MoO_3_ reduction initiates with the hydrogen adsorption onto terminal oxygen atoms and in the layer at oxygen atoms of corner-sharing MoO_6_ units along the [1 0 0] direction [25]. The MoO_3_ reduction to MoO_2_ by hydrogen is an exothermic reaction with the release of heat and consequent local temperature increase [27].

On the other hand, the morphology generally affects MoO_3_ performance, and this is because MoO_3_ has a unique structure. For the most part, the different morphologies of MoO_3_ and MoO_3_-based materials determine their applications. For example, MoO_3_ nanorods usually have porous structures that will improve charge and mass migration, which can be used for energy storage devices. MoO_3_ nanotubes are regarded as one of the most promising architectures for ion batteries and gas sensors because of their large specific surface area, low density, and superior transport. Nanoflowers and nanospheres can make the surface area bigger and give it more active sites, which can improve its performance in areas like sensing, energy storage conversion, and catalysis [28,29,30].

This article aims to briefly review and highlight the main interesting and applicable properties and the latest advances in technological applications of MoO_3_ and MoO_3_-based materials in gas sensors, as anode and cathode materials for lithium-ion batteries, and water pollution treatment using adsorption and photocatalysis techniques, presenting the most relevant and new information on heterostructures, metal doping, and non-stoichiometric MoO_3−x_.

## 2. Air Pollution and Gas Sensing Applications

Air pollution is considered one of the major 21st-century challenges due to the rapid growth of industries and automobiles, which cause high air pollutant emissions [31]. Among the various pollutants, volatile organic compounds (VOCs) are mostly obtained from exhaust gases, fuel combustion, and transportation. People can get headaches, nausea, convulsions, and commas if exposed to a certain VOC concentration, and many carcinogens can also harm the liver, kidneys, brain, and neurological system [32]. For this reason, there is a growing need to design and fabricate sensing devices with high response and selectivity, low fabrication costs, and a flexible mechanical nature to control air quality [31]. Smart gas sensors are currently widely used in air quality monitoring (humidity or oxygen sensing in car cabins or houses), biosensors for diagnosing a variety of diseases, and leak detection of harmful and explosive gas emissions in industries, which may lead to severe accidents and health hazards [33].

Generally, a gas sensor is a device or chemical sensor that typically consists of a transducer with an active layer that transforms the analyte species information into electrical signals from many detection principles, such as changes in conductivity, resistance, absorbance, etc., in the presence of gas molecules. As electrical properties change, the gas analyte interacts as a donor or an acceptor of charge carriers, changing the material’s resistivity. This resistance change is related to the type of majority carriers present and also to the oxidizing or reducing nature of the gas [33]. The receptor function of the gas sensing material, its converting function, and its utility function influence gas-sensing properties, particularly sensitivity. The receptor function is concerned with the oxide capacity of the surface to interact with the detecting gas molecules, while the transforming function is concerned with the signal conversion to an electric signal, which is Induced by the chemical interaction between the oxide surface and the detecting gas. The utility function is associated with the availability of the inner oxide grains for the molecules of the detecting gas [34].

Among the existing gas sensors, the metal oxide-based ones, known as resistive (Chemi-resistors) or hot-wire sensors, present interesting advantages in their good reliability and simple implementation for real-time monitoring of various gases as VOCs [35,36]. These advantages are attributed to their unique merits, such as high structural stability, portability, adjustable electrical properties, reduced grain size, surface morphologies, easy production, simple operation, and ease of system integration [33,37,38,39]. For this reason, a lot of research efforts have been invested in this kind of gas sensor since the first fundamental idea demonstration in 1962 [39]. This sensor type shows itself to be sensitive to environmental chemical reactions, and due to its stability to operate in harmful environments, it can compete with other sensor types in terms of reliability, sensitivity, and accuracy, having received much more attention since the second half of the 20th century [36].

**Figure 4:**
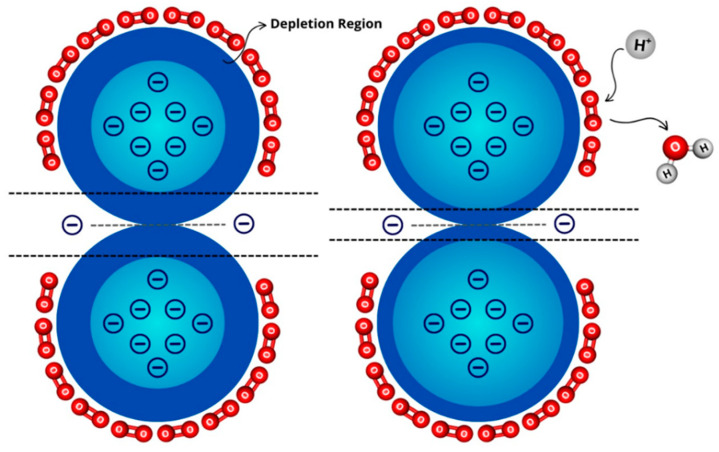
Scheme of the reaction mechanism of MoO_3_ materials exposed to hydrogen gas. Based on Ref. [36].

The molybdenum trioxide (MoO_3_), as a wide band gap n-type and layered structured semiconductor, has been extensively investigated and applied in gas sensors, where it has been found to be sensitive to different gases such as H_2_S [40], NO_2_ [31,41,42], NO [31], H_2_ [36], CO [43], CO_2_ [44], C_2_H_5_OH [34,35,45,46], NH_3_ [47], trimethylamine [48,49], acetone, 1-butylamine [37], triethylamine [38,50,51], formaldehyde [52], water/humidity sensor [53] and glucose [54], with detection limits often in ppm range [31,35,36]. A reaction mechanism generality for sensing the gases is shown in Figure 4. Initially, the oxygen molecules are absorbed at the surface of MoO_3_, adding the depletion region, i.e., the electron-depleted space charge region in which the absorbed oxygen molecule captures electrons from the MoO_3_-conduction band, as a result of its high electron affinity (Figure 4, left panel). Then, in the presence of a gas sensing, for instance H_2_, the previously absorbed oxygen molecules react with the H_2_ gas and subsequently release the captured electrons from the MoO_3_ conduction band. This reduces the depletion region and increases MoO_3_ conductivity (Figure 4, right panel). 

**Figure 5:**
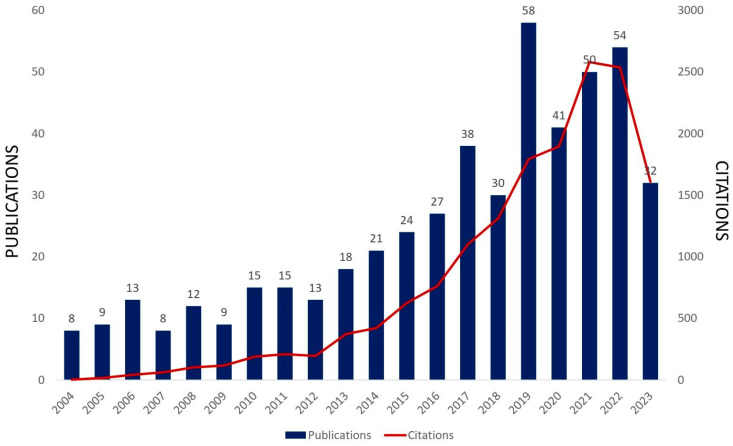
Published scientific papers and citations directly related to MoO_3_ and MoO_3_-based systems applied to gas sensors in the last 20 years (Web of Science search with MoO_3_ and gas sensors keywords, 2023 ongoing, Access on 9 October 2023).

According to Figure 5, there has been an increasing trend in the last 20 years in papers directly related to MoO_3_ and MoO_3_-based systems applied to gas sensors. A summary of recent research on MoO_3_ and MoO_3_-based systems in gas sensing applications in the last seven years is presented in Table 1.

**Figure 6:**
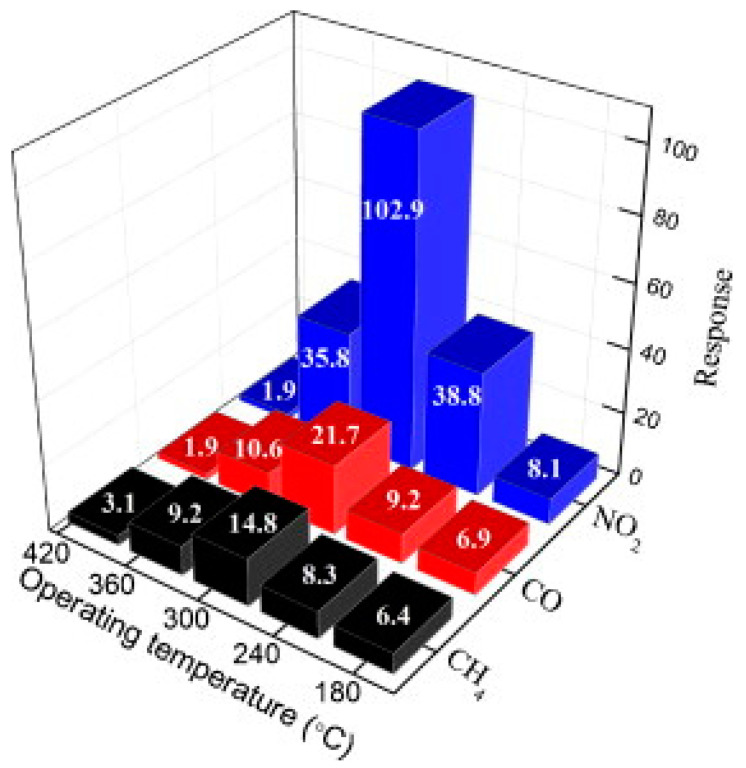
Responses of MoO_3_ nanorods to NO_2_, CO, and CH_4_ gases at 40 ppm and different temperatures. Figure reproduced with permission of [41] Copyright 2012, Elsevier.

Moreover, this oxide has gained significant interest as an electrode material for electrochemical sensors owing to its high electrochemical activity, high dielectric constant, and good gas molecule adsorption [55]. Because of their charge carrier concentration modulation in response to oxidizing and reducing gases, molybdenum oxides are employed in conductometric gas sensing [1]. The performance of these MoO_3_-based gas sensors depends on gas type sensing (oxidizing or reducing gases), as different reaction mechanisms occur between the molybdenum oxide surface and the target gas molecule, leading to a change in the conductivity of MoO_3_ (Figure 6). Interestingly, the MoO_3_ sensing mechanism is considered to be different from those of other common n-type semiconductors (e.g., SnO_2_ or ZnO). It is proposed to be a lattice oxygen reaction (catalyst property) rather than a traditional surface-chemisorbed oxygen mechanism due to its crystalline-layered structure [37,56]. The lattice oxygen reaction mechanism indicates that the decrease in resistance is related to the free electrons created from the oxygen vacancies by the partial reduction of Mo^6+^ to Mo^5+^ ions via the catalytic oxidation reaction between the reducing agents and the lattice oxygen [37]. In this process, shear structures are formed by removing lattice oxygen at the active oxide surface, which is related to the ease of rearrangement of polyhedral [56].

Despite its attractive properties, the unsatisfactory MoO_3_ electrical conductivity limits its electrochemical performance [55]. To improve this, MoO_3_ functional and surface modifications, morphological and sizing adjustments by crystal growth interference exposing different crystal planes, doping elements, loading noble metals, and composite synthesis have been considered as interesting options [43,57,58]. The chemisorption activation energy can be effectively decreased with suitable metal doping [32]. These metal elements work as catalytic activity centers that change the way electrons move inside the sensor and make it better at detecting gases, obtaining a considerable response, remarkable selectivity, reliable stability, and a rapid recovery rate [32,38,43]. The heterostructures interfere with properties such as carrier concentration, grain boundary barrier, depletion layer, and energy band, improving gas sensing performance [32]. Several types of research have then been performed using nanocomposites applied to different gases, for instance, carbon monoxide, triethylamine, trimethylamine, formaldehyde, 1-butylamine, hydrogen, benzene, toluene, xylene, glucose, ethyl acetate, and ethanol, like Cr-MoO_3_ [38], Zn-MoO_3_ [43], Pt-MoO_3_ [36,52], Au-MoO_3_ [37,49,59], TiO_2_-MoO_3_ [34], MoO_3_-ZnO [46], SnS-MoO_3_ [51], NiO-MoO_3_ [60], PVP-MoO_3_ [54], and MoO_3_ nanowires [61].

Particularly, the satisfactory high reactivity and thermodynamic stability of some reducing agents make the α-phase MoO_3_ a standout option for this application [57]. The layered structure associated with α-MoO_3_ increases the Mo^5+^ pentavalent ions, which have a strong affinity for oxygen [30]. The electrical conductance of non-stoichiometric MoO_3−x_ is higher than that of stoichiometric metal oxide due to enhanced charge carrier mobility associated with the presence of low-valent Mo ions, which is the sensing principle of MoO_3_ [37,56]. One way to synthesize non-stoichiometric MoO_3−x_ materials and consequently improve the selectivity and sensing performance of these sensors is to introduce oxygen vacancies into the lattice, causing structural defects. The presence of these vacancies plays a significant role in the surface charge distribution under thermal excitation. Then, the band gap can be adjusted by these lattice vacancy insertions, creating gap states. These oxygen vacancies also act as an adsorbing center with a high affinity toward gases at high temperatures. Moreover, the environmental oxygen molecules adsorb spontaneously on the surface of vacancy sites, leading to the formation of more active oxygen species that trap free electrons next to the surface area. At lower temperatures, these oxygen vacancies are not reactive, leading to high resistance in the channel. Otherwise, higher operating temperatures allow abundant thermal energy to exceed the activation energy barrier to accomplish the surface reaction, resulting in an increasing free charge carrier density close to the conduction [56].

The presence of oxygen vacancies can often cause several interface and surface physicochemical changes, including the formation of a new donor level in the forbidden band and an increase in the concentration of available carriers [57]. The electrical conduction of MoO_3_ is mostly dependent on the free electrons available in its conduction bands. The defect concentration, such as an oxygen deficit, significantly impacts the free-electron intensities in these materials [1]. Additionally, under the influence of oxygen vacancies, the surface lattice structure can change from a stable state to a metastable state, resulting in the formation of an unsaturated coordination metal atom at the vacancy as the center of free electrons and opening up a new pathway for the interaction of electrons with target gas molecules. The amount of surface chemisorbed oxygen increases as a result of plentiful oxygen vacancies acting as active sites that can also provide abundant unpaired electrons acting as electron donors, boosting the adsorption activity [57].

**Table 1:** MoO_3_ and MoO_3_-based systems used in gas sensing applications. Optimal sensing: ratio between the resistance of the target gas and the reference gas (N_2_). Optimal temperature: working temperature.

Material	Morphology	Synthesis Method	Working Temp. (°C)	Gas (Conc.)	Optimal Sens. (%)	Optimal Temp. (°C)	Reference
α-MoO_3_	Thin Films	Pulsed laser deposition	100 and 200	NO_2_ (10 ppm)	25	100	[31]
α-MoO_3_	Nanobelts	Hydrothermal	(250–400)	C_2_H_6_O (800 ppm)	173	300	[35]
α-MoO_3_	Nanobelts	Hydrothermal	(100–400)	1-butylamine (100 ppm)	~90	340	[37]
α-MoO_3_	Nanorods	Hydrothermal	(100–350)	Triethylamine (100 ppm)	~115	200	[38]
α-MoO_3_	Nanoflowers/nanosheets	In situ oxidation	(100–350)	H_2_S (10 ppm)	~38	250	[40]
α-MoO_3_	Large-sized	Vapor-phase transport	(50–125)	NO_2_ (10 ppb)	2.3	100	[42]
α-MoO_3_	Hierarchical microflower	Thiourea-assisted hydrothermal	(120–380)	CO (50 ppm)	8.02	260	[43]
α-MoO_3_	Nanoparticles	Thermal evaporation	(150–250)	CO_2_ (150 ppm)	15	250	[44]
α-MoO_3_	Hierarchical nanofiber-assembled	Hydrothermal	(50–350)	C_2_H_6_O (400 ppm)	24	300	[45]
α-MoO_3_	Hierarchical nanosheet-assembled	Hydrothermal	(50–350)	C_2_H_6_O (400 ppm)	32	300	[45]
α-MoO_3_	Nanorods	Thermal evaporation under vacuum	RT ^a^	NH_3_ (100 ppm)	886	RT ^a^	[47]
α-MoO_3_	Porous nanosheets	Solvothermal	50, 90, 133, 172, and 217	Trimethylamine (10 ppm)	51.47	133	[48]
α-MoO_3_	Nanorods	Hydrothermal	(100–350)	Triethylamine (100 ppm)	153.36	200	[50]
α-MoO_3_	Nanorods	Hydrothermal	250	Triethylamine (50 ppm)	73.46	250	[51]
α-MoO_3_	Nanowires	Hydrothermal	(200–320)	Triethylamine (500 ppm)	~4150	280	[61]
h-MoO_3_	Nanosheets	Dispersion	RT ^a^	NH_3_ (0.3 ppm)	3.78	RT ^a^	[39]
α-MoO_3−x_	Thin films	Chemical vapor deposition	(160–270)	NO_2_ (10 ppm)	56	250	[56]
α-MoO_3−x_	Thin films	Chemical vapor deposition	(160–270)	H_2_S (10 ppm)	18	250	[56]
Pt-α- MoO_3_	Nanoparticles	Dispersion	(170–300)	H_2_ (500 ppm)	18	260	[36]
Au-α-MoO_3_	Nanoparticles/Nanobelts	Hydrothermal	(100–400)	1-butylamine (100 ppm)	~300	240	[37]
Zn-MoO_3_	Hierarchical microflower	Thiourea-assisted hydrothermal	(120–380)	CO (50 ppm)	31.23	240	[43]
Au-MoO_3_	Nanoparticles/nanobelts	Hydrothermal + Dispersion	(180–300)	Trimethylamine (50 ppm)	70	280	[49]
Pt-MoO_3_	Nanobelts	Hydrothermal + Dispersion	RT ^a^	HCHO (200 ppm)	39.3	RT ^a^	[52]
Au-MoO_3_	Nanoparticles/hollow spheres	Surfactant-modified approach	(217–330)	Toluene (100 ppm)	17.5	250	[59]
Au-MoO_3_	Nanoparticles/hollow spheres	Surfactant-modified approach	(217–330)	Xylene (100 ppm)	22.1	250	[59]
Cr_2_O_3_-α-MoO_3_	Nanorods	Hydrothermal	(100–350)	Triethylamine (100 ppm)	150.25	200	[38]
α-MoO_3_-ZnO	Nanoparticles/nanobelts	Hydrothermal	(100–300)	C_2_H_6_O (400 ppm)	19	250	[46]
α-MoO_3_-SnS	Nanotubes	Hydrothermal	250	Triethylamine (50 ppm)	36.06	250	[51]
NiO-α-MoO_3_	Core-shell nanorods	Hydrothermal + Dispersion	(150–350)	(100 ppm)	34.91	250	[60]
h-BN-α-MoO_3_	Nanowires/2D material	High-temperature pyrolysis	(200–320)	Triethylamine (500 ppm)	8616	260	[61]

^a^ Room Temperature.

Compared to other metal oxides, α-MoO_3_ is considered a potential material for detecting triethylamine (TEA), since it is an excellent catalyst for dissociating the carbon-nitrogen bonds of organic amines and specifically interacts with TEA molecules due to strong interactions between acidic α-MoO_3_ surfaces and basic TEA and the lower activation energy for the carbon-nitrogen bonds compared to other chemical bonds. The Cr-doping into the α-MoO_3_ lattice promoted the Mo^5+^ existence due to the similar ionic radii of Mo^+6^ and Cr^+3^, resulting in higher responses to TEA [38]. The 2D α-MoO_3−x_ sensors also presented dual gas sensing characteristics, with excellent sensitivity and selectivity toward H_2_S (10 ppm) and NO_2_ (10 ppm) at 250 °C, compared to other gases such as H_2_ (1000 ppm), CO (1200 ppm), CO_2_ (1000 ppm), and CH_4_ (10,000 ppm). The highly electrophilic nature of NO_2_ was shown to contribute to its strong oxidizing properties and to be extremely reactive toward the intrinsic oxygen vacancy sites on the α-MoO_3−x_ surface. Upon H_2_S exposure, the surface oxygen vacancy sites of MoO_3−x_ showed an interaction with H_2_S gas molecules and released charge carriers, resulting in defect states within the energy gap. Hence, the sensor presented an n-type behavior of MoO_3−x_ toward a reducing gas [56].

**Figure 7:**
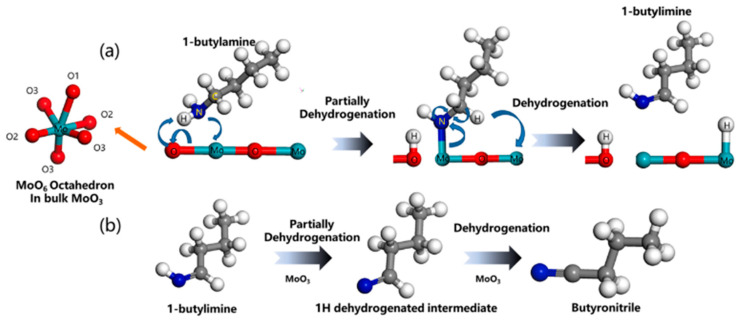
Schematic diagram illustrating the 1-butylamine sensing mechanism on the MoO_3_ surface showing the dehydrogenation pathway of (**a**) 1-butylamine and (**b**) 1-butylimine. Figure reproduced with permission of [37] Copyright 2021, Elsevier.

When compared to alcohols (methanol, ethanol, and n-butanol), acetone, and pyridine, MoO_3_ and Au-MoO_3_ nanocomposites were very selective for 1-butylamine. The unique layered structure of MoO_3_ and the type of connection within the 1-butylamine are responsible for its strong selectivity. Among these gases (1-butylamine, methanol, ethanol, n-butanol, and pyridine), both n-butanol and 1-butylamine molecules have a butyl group, which is a relatively strong electron-donating group compared with methyl and ethyl groups. The nitrogen atom in 1-butylamine and the oxygen atom in n-butanol have lone pair electrons that act as electron donors to form bonds. Such structure is preferentially absorbed onto Mo ions that are considered Lewis-acid sites (see Figure 7). The Au-MoO_3_ nanocomposites also showed a high response to NH_3_ and NO_2_, associated with the MoO_3_ and N atom interactions [37].

Moreover, the α-MoO_3_ surface showed to be beneficial for the adsorption of H_2_O molecules because of the pentacoordinated Mo atoms introduced by the interchain interactions breaking, which possessed the Lewis acid character of an electron pair acceptor. The adsorption of H_2_O molecules on these Mo sites occurs mainly to balance the electrostatic force around the Mo atoms, allowing the obtaining of a robust humidity sensor presenting a fast response and high sensitivity within the wide sensing range of 10–90% relative humidity (RH) [53].

## 3. Lithium-Ion Batteries

**Figure 8:**
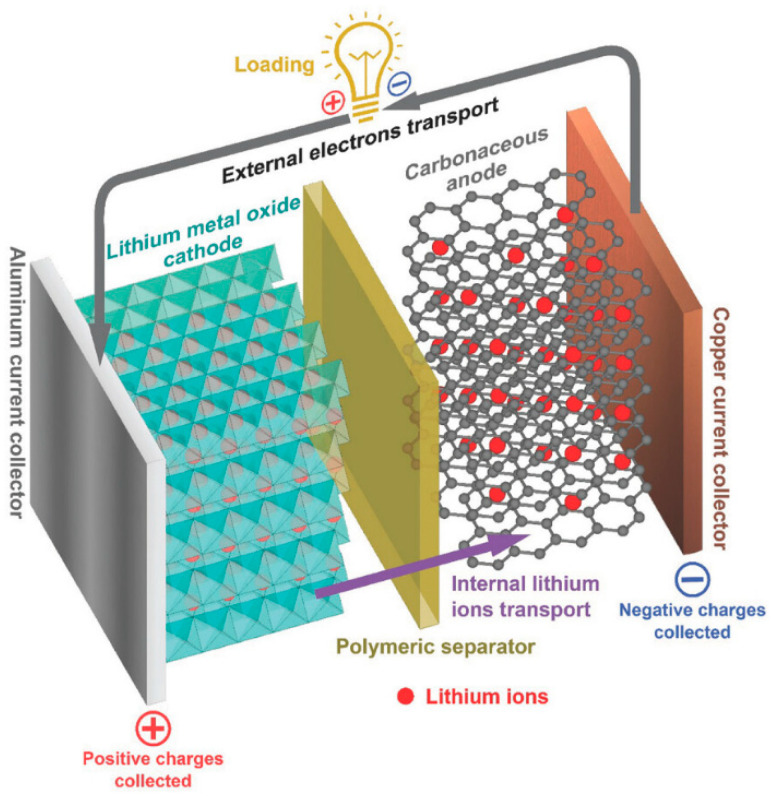
A schematic diagram illustrates an external load applied to both electrodes. This is the discharging process of a lithium-ion battery. Figure reproduced with permission of [68] Copyright 2017, Wiley.

Lithium-ion batteries (LIBs) have become one of the most important and practical energy storage systems for power sources due to their power density, higher energy, longer lifespan, high operating voltage, excellent rechargeability, stable cycle performance, no memory effects, considerable environmental friendliness, and beneficial safety [24,62,63,64,65,66,67]. The LIBs have been researched for use in a variety of electronic devices, including laptops and cell phones, as well as electric and hybrid cars [24,62,63]. Two solid-state electrodes (the cathode and anode), an electrolyte containing Lithium ions, a spacer polymer, and two current collectors typically make up Lithium batteries (Figure 8). Each component of the battery is crucial; for instance, the separator prevents a short circuit inside the cell, and the electrode material determines how much energy they can store. Thus, research on electrode material property election is fundamental to the storage efficiency of the battery. 

Nowadays, graphite dominates as an anode material in LIB because of its advantages of great abundance and low cost. However, its lower and limited theoretical Li-storage capacity (372 mA·h·g^−1^) and slower discharge potential are insufficient to fulfill the increasing energy-consuming demands [24,63]. Alternatively, molybdenum oxides have been researched as anode materials for lithium-ion batteries, benefiting from their higher specific capacities, acceptable cost, non-toxicity, and high stability [24,62,68]. Interestingly, MoO_3_ is also a potential Li-ion battery material that can function as a cathode [69,70,71,72]. For example, Wang and co-workers used an ammonolysis process to modify the crystal structure and consequently enhance the electrochemical performance of MoO_3_ nanobelts when tested as cathodes for Li-ion batteries [70]. The modified nanobelts exhibited a capacity of 250 A·h/kg in the potential window of 1.5–3.5 V. The unfavorable operating voltage of reduced Mo oxides makes them possible for use as either cathodes or anodes. Typical redox voltages for Mo oxides lie in a regime that hinders maximizing energy density when they are paired with higher-voltage cathodes or lower-voltage anodes [73]. Due to the wide range of oxidation states (from +6 to +2), they are promising as both positive (cathode) and negative (anode) electrodes in electrochemical cells [74].

One of the most important and interesting properties of molybdenum oxides is their intercalation ability with a wide range of ion sites, which is possible if the host material layers or tunnels have weak binding [75]. This is a consequence of the weak Van Der Waals force between the stacked sheets along the [0 1 0] direction [68]. The α-MoO_3_ characteristic layer structure forms tetrahedral and octahedral holes and extension channels, leading to more diffusing channels and embedding sites for Li^+^ ions [75].

**Figure 9:**
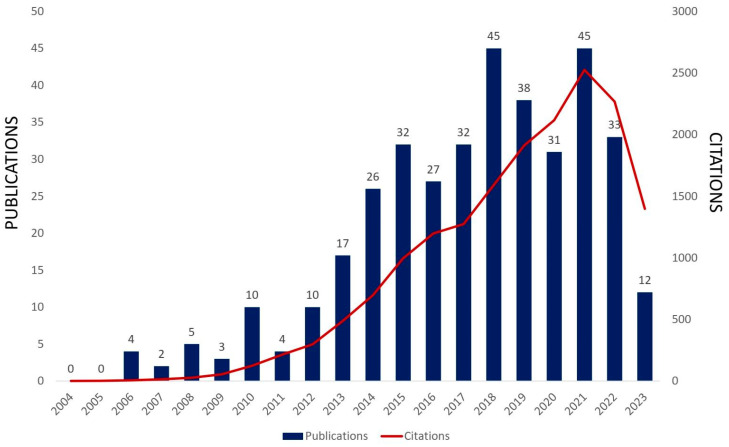
Published scientific papers and citations directly related to MoO_3_ and MoO_3_-based systems applied to LIBs in the last 20 years (Web of Science search with MoO_3_ and lithium-ion batteries keywords, 2023 ongoing, Access on 9 October 2023).

Molybdenum oxides can experience redox reactions that are highly reversible, such as Mo^6+^/Mo^4+^. Theoretically, these reactions produce a high energy density of 745 W·h·kg^−1^ because they permit numerous electron exchanges per molybdenum redox center and can load up to 1.5 Li per molybdenum atom [1,68]. Additionally, the potential Li-storage capacities of the MoO_3_ and MoO_2_ presented have high theoretical specifications: 1117 mA·h·g^−1^ and 838 mA·h·g^−1^, respectively. These molybdenum oxides weak electrical conductivity (MoO_3_ = 9.6 × 10^−7^ s·cm^−1^, MoO_2_ = 8.8 × 10^−5^ s·cm^−1^) and slow diffusion kinetics of Li^+^ ions, however, are often their limitations [24]. In addition, the molybdenum oxides undergo a significant volume expansion of around 100–250% during insertion and extraction. This causes internal tensions, electrode pulverization, and loss of interparticle contact, which leads to electrical disconnection [24,63,75]. Poor rate performance, severe capacity fading, and suboptimal cycling stability are the results of these problems [63,75]. However, there has been an increase in the past 20 years in papers specifically about MoO_3_ and MoO_3_-based systems used for lithium-ion batteries (Figure 9), with many studies concentrating on solving these difficulties because of the benefits of these materials. Table 2 lists recent studies on MoO_3_ and MoO_3_-based systems for LIBs.

To get around the problems with molybdenum oxides and get stable cycling with a higher reversible capacity and rate performance, researchers have looked into a number of effective strategies, especially to lower the diffusion barrier and band gap and shorten the electron and Li^+^ diffusion channels [76]. When nanostructures like nanosheets, nanoparticles, nanobelts, and nanowires are made, it is easier for electrolyte ions to reach the active sites [75,76]. On the other hand, bandgap and diffusion barriers have been lowered via vacancy engineering and heteroatom doping. The creation of oxygen vacancies has been suggested as a way to improve lithium storage efficiency since they can dramatically boost ion diffusion kinetics, increase electrical conductivity, and provide more active sites for redox reactions [76]. Lithium ions can be inserted and removed during charging and discharging cycles at the vacant sites in the MoO_3_ crystal intralayer and interlayers [68].

Free electrons present in the MoO_3_ conduction bands are primarily responsible for its electrical conductivity, and the intensities of these free electrons in such material are significantly influenced by the concentration of defects, such as oxygen deficiency [1]. By electron-proton co-doping between low-work-function metals and MoO_3_ in a deionized water environment, it is possible to create oxygen vacancies. The hydrogen doping preferentially chooses symmetric oxygen to generate the unstable OH group, which distorts the MoO_3_ lattice. By releasing this unstable OH group as H_2_O into the solution, MoO_3_ loses oxygen from its lattice and forms oxygen vacancies [76]. Two-dimensional (2D) MoO_3_ materials have also attracted great interest due to the shortened lithium-ion transportation path and released stress concentration. These materials also provide more sites for lithium insertion and can significantly increase lithium storage capacity [75].

In addition, MoO_3_ nanostructured materials engineered by carbon coatings would surely bring advantages for electrochemical performances due to the potential capability of the synergetic effects of each component [24]. Carbonaceous materials such as graphene, amorphous carbon, carbon nanotube (CNT), and graphite can improve structural stability, compensate for the low electrical conductivity of MoO_3_ anodes, and accommodate the large strain induced by Li-ion diffusion during cycling [62]. Conversion reactions are involved in the lithiation/delithiation mechanism for the layered MoO_3_ [24,63]:

(1) $${MoO}_{3}+x{Li}^{+}+xe^{-}↔{Li}_{x}{MoO}_{3}$$

(2) $${Li}_{x}{MoO}_{3}+\left(6-x\right){Li}^{+}+\left(6-x\right)e^{-}↔3{Li}_{2}O+Mo$$

The rutile-type structure of MoO_2_ features a high local defect concentration that changes the atomic arrangement, facilitating the transport and storage of Li^+^ ions. Unlike MoO_3_ and similar to the graphite mechanism, MoO_2_ follows just a one-step reversible reaction [24]:

(3) $${MoO}_{2}+x{Li}^{+}+xe^{-}↔{Li}_{x}{MoO}_{2}$$

**Table 2:** MoO_3_ and MoO_3_-based systems used in LIBs applications.

Material	Morphology	Synthesis Method	Current Density (mA·g^−1^)	Li Ion Capacity (mA·h.g^−1^) ^a^	Cycles	Initial Coulombic Efficiency (ICE) (%)	Reference
α-MoO_3_	Sheets	Thermal plasma	3000	700	200	70	[75]
α-MoO_3_	Nanobelts	Hydrothermal	100	787	100	70.6	[77]
α-MoO_3−x_	Nanobelts	Mechanical grinding	500	820	200	UD ^b^	[78]
N-α-MoO_3−x_	Nanoflowers	NR ^c^	100	1261	450	81.2	[76]
Carbon-α-MoO_3_	Nanoparticles/Nanofibers	Electrospinning	500	801.1	200	~98	[4]
Carbon-α-MoO_3_-MoO_2_	Nanoribbons/Nanoparticles	Solid-phase	100	840	100	52	[24]
CNT-α-MoO_3_	Nanofibers/Nanoplates	Electrospinning	1000	972	100	62.7	[62]
α-MoO_3_-SnS_2_	Nanorods/Nanosheets	Hydrothermal	60	568.2	100	92.7	[79]
Carbon-MoO_2_	Nanoplate-like	CO_2_ oxidation	50	323	300	UD ^b^	[80]
α-MoO_3_-TiO_2_	Nanobelts	Hydrothermal	400	935.8	400	73	[81]
α-MoO_3_-NiO	Flower-like microspheres	Hydrothermal + Coverage	100	944	100	81	[82]
Carbon-α-MoO_3_-SnO_2_	Nanoflakes	Chemical Vapor Deposition + Dispersion	200	1020.5	200	62.8	[83]
α-MoO_3_-RGO	Nanoparticles	Ultrasonication	500	568	100	UD ^b^	[84]
CNT-SiO_2_-α-MoO_3_	Cactus-like	Self-assembly and in situ carbonization	1000	700	500	99.8	[85]
Fe_2_O_3_-TeO_2_-MoO_3_	Nanoparticles	Molten	1000	463.2	800	47.4	[86]
Carbon-SnO_2_-MoO_3_	Nanoparticles/Nanosheets	Hydrothermal	200	1338.3	300	70	[87]
h-MoO_3_-Grafene	Microrods	Scalable precipitation	2000	665	300	63	[88]
h-MoO_3_-GO	Microrods	Scalable precipitation	1000	789	100	65	[88]
SnO_2_-MoO_3_-Graphene	Nano-grain/Sheet-like	Hydrothermal	200	1522.5	250	72.2	[89]
SnO_2_-MoO_3_-CNT	Nanoparticles/Nanotubes	Hydrothermal	200	1372.2	280	80.9	[90]
Carbon-α-MoO_3_	Nanoparticles	Solution combustion	100	668	200	UD ^b^	[91]
α-MoO_3_-Fe_2_O_3_	Micro-octahedrons	Thermolysis	200	1218	350	81.9	[92]
α-MoO_3_-MoO_2_-g-C_3_N_4_	Nanosheets	Hydrothermal + Freeze-Drying	500	992	100	72	[93]

^a^ Value after the cycling test. ^b^ UD: Unavailable Data. ^c^ NR: Numerical Approach.

## 4. Water Pollution Treatment: Adsorption and Photocatalysis

Global industrial development has led to undesirable environmental problems, and one of the worst is water pollution. Among the water pollutants, heavy metal ions are harmful contaminants because of their high toxicity and mutagenicity/carcinogenicity potential, even at very low concentrations [94,95]. Organic toxic substances and several heavy metals are discharged into the environment without appropriate treatment. These pollutants originate from the paper, textile, food, pharmaceutical, printed circuit board manufacturing, and solar cell industries [15,96,97]. Because of this, a high number of studies have focused on the development of more efficient, low-cost, easy-to-operate, environmentally friendly, and reusable materials for water treatment. The adsorbents and the adsorption process have become two of the principal methods to remediate this problem. However, the search for new, low-cost, and more effective sorbents is still a challenge worldwide [95,97,98].

**Figure 10:**
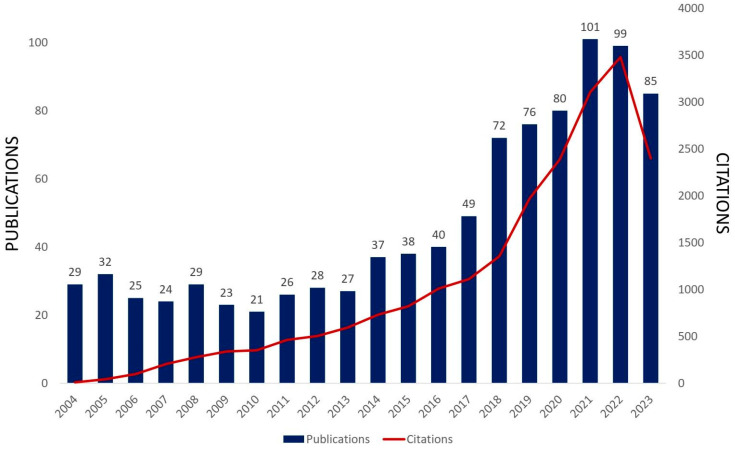
Published scientific papers and citations directly related to MoO_3_ and MoO_3_-based systems applied to adsorption in the last 20 years (Web of Science search with MoO_3_ and adsorption keywords, 2023 ongoing, Access on 9 October 2023).

Molybdenum-based materials have been indicated as efficient materials for wastewater treatment due to their attractive and unique adsorptive, catalytic, optical, and electronic properties, in addition to their high mechanical, thermal, and chemical stability [97]. According to Figure 10, an increasing trend in the last 20 years in papers directly related to MoO_3_ and MoO_3_-based systems applied to adsorption has been verified.

**Figure 11:**
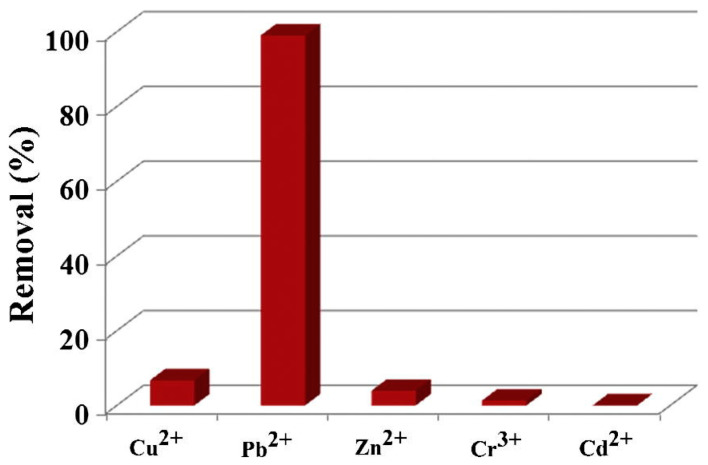
Removal efficiency of metal ions Cu^2+^, Pb^2+^, Zn^2+^, Cr^3+^, and Cd^2+^ removed by the α-MoO_3_ nanosheet array system. Figure reproduced with permission of [94] Copyright 2017, Elsevier.

Oxygen vacancies are often formed on the surface with a negative zeta potential, and consequently, strong electrostatic adsorption between MoO_3−x_ and cationic dyes is observed [98,99]. In Table 3, recent studies on MoO_3_ and MoO_3_-based systems in the adsorption process are shown. Interestingly, these materials present high adsorptive capacity, especially towards cationic dyes such as methylene blue (MB) [22,97,98,100,101,102,103], crystal violet (CV) [101], and malachite green (MG) [101]. In addition, heavy metal ions such as Pb^2+^ [94,95,97,104], Cr^3+^ [102], Cu^2+^ [104], Mn^2+^ [105], Cr^6+^ [105], and aromatic sulfur compounds like thiophene [106] and dibenzothiophene [106] are removed by the electrostatic adsorption interactions. Removal efficiency of some metal ions with an a-MoO_3_ nanosheet array system is studied by Yunying Wu et al. [94], showing a higher adsorption capacity toward Pb^2+^ from an aqueous solution than Cu^2+^, Zn^2+^, Cr^3+^, and Cd^2+^ (see Figure 11). The enhanced performance in Pb^2+^ removal can be attributed to the electrostatic adsorption interactions between oxygen-containing functional groups and Pb^2+^, besides the partial formation of coordination bonds between oxygen groups and Pb^2+^ and also the formation of a new substance, the PbMoO4 precipitate [94,97], from a solid-liquid interfacial reaction as shown in Equation (4) [94]:

(4) $${MoO}_{3}+H_{2}{O+{Pb}^{2+}\to PbMoO}_{4}+2H^{+}$$

Kedves et al. observed that the α-MoO_3_ adsorption capacity has a direct relationship with the cationic group quantity [101]. The presence of imine, two amino, and thiol groups in the molecule of methylene blue favored faster adsorption by the α-MoO_3_-based system. On the contrary, crystal violet with three amine functional groups, malachite green with only two, and rhodamine B with two amine groups with a carboxylic and an ether group added resulted in reduced adsorption efficiency. Thus, the adsorption effectiveness declines as the number of cationic groups decreases. The presence of more positively charged functional groups in methylene blue tends towards greater bond formation with the negatively charged α-MoO_3_ surface.

For anionic dye adsorption, an interesting strategy is to produce composites by intercalating the favorable layered structure of MoO_3_ with active sites charged positively. Wang et al. [107] prepared the Al_13_-3.34%@MoO_3_ composite by intercalating polycationic Al_13_ into an extensive airspace of MoO_3_. The anionic dye methyl orange (MO) was rapidly adsorbed due to the strong electrostatic interaction between the cation Al_13_ and the anion MO.

MoO_3_ materials are also effective in removing metallic ions such as Pb^2+^. The electrostatic interaction between O-containing functional groups and Pb^2+^, as well as the partial creation of coordination bonds between oxygen groups and Pb^+2^, govern the process of Pb^2+^ removal by MoO_3_. The thermodynamic parameters [97] suggest that the Pb^2+^ removal on α-MoO_3_ is spontaneous and endothermic, and the Pb^+2^ removal matches quite well with the Langmuir isotherm. The pH solution interferes with the Pb^+2^ adsorption process. In the range (2.0–8.0), there are three forms of lead species: Pb^2+^ (dominant at pH < 6.0), Pb(OH)^+^ (formed from the Pb^2+^ hydrolysis started at pH = 3.7 and dominant at pH > 7.5), and Pb(OH)_2_ (formed from the Pb^2+^ hydrolysis started at pH = 6.8) [108]. The Pb^2+^ removal slightly increased upon increasing the pH from 2.0 to 5.0, and the removing rate reached its highest value at pH = 5.0. However, it slightly decreased when the pH increased from 7.0 to 8.0. The results suggested that the electrostatic interactions occurred between α-MoO_3_ and Pb^2+^/Pb(OH)^+^ ions. Thus, α-MoO_3_ exhibited a high removal capacity over a broad pH range due to its negative surface charge in this pH interval, presenting high selectivity toward Pb^2+^ compared to Cu^2+^, Zn^2+^, Cr^3+,^ and Cd^2+^ ions [94].

**Figure 12:**
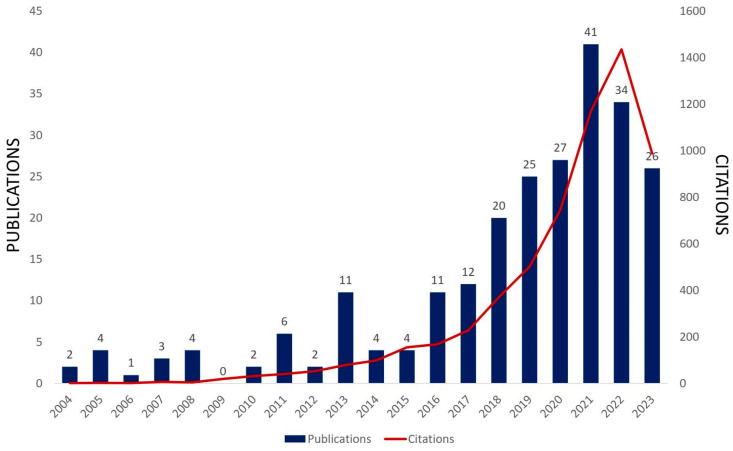
Published scientific papers and citations directly related to MoO_3_ and MoO_3_-based systems applied to photocatalysis in the last 20 years (Web of Science search with MoO_3_ and photocatalysis keywords, 2023 ongoing, Access on 9 October 2023).

Molybdenum trioxide (MoO_3_) is an n-type semiconductor that has been used as an active photocatalyst because of its effective absorption of the majority of UV light and partial visible light absorption, with a wide optical band gap (2.8–3.6 eV) that varies depending on the growth conditions and synthesis method [18,72,109]. An increasing trend in the last 20 years in papers directly related to MoO_3_ and MoO_3_-based systems applied to photocatalysis is verified in Figure 12. Recent studies on MoO_3_ and MoO_3_-based systems used in photocatalytic applications are summarized in Table 4. Photocatalytic applications benefit from the multiple Mo valence states, chemical stability, non-toxicity, ability to transport holes, and high aqueous medium stability [110]. In particular, MoO_3_ materials have a strong oxidation capability in the photocatalytic process due to their low valence band level [19]. However, MoO_3_ presents limited photocatalytic effects due to its fast electron-hole recombination and limited visible light absorption [111,112,113]. To solve these issues, these semiconductors are doped, which is considered a promising strategy to improve photocatalytic performance [111,114]. The heterojunction formed by the different individual band gaps creates intermediate energy levels in the gap region, which facilitate the electronic transition and provide efficient separation of electron-hole pairs and recombination retardation [112]. This occurs through a rectifying effect at the heterojunction interface and promotes the enhancement of the photocatalytic reactions. The heterostructures also provide large interfacial areas for the occurrence of redox reactions [112]. Thereby, heterostructured systems doped with Fe_2_O_3_ [15,111], rGO [15], SnO_2_ [115], Cu_2_S [116], WO_3_ [110,115], CdTiO_3_ [117], SiO_2_ [118], g-C_3_N_4_ [58,112,119], NiO [120], polypyrene [121], etc., and also metal doping with Ag [118] were synthesized in the last years for photocatalytic applications such as dye and antibiotic degradation and heavy metal reduction (Cr^6+^), showing excellent performance for RhB [58,112,118], MB [15,115,116,120,122], tetracycline [111], acetaminophen [121], and metronidazole photodegradation under visible light irradiation [118], besides aspirin degradation under UV irradiation [117] and MB and 2,4-dichlorophenol under sunlight irradiation [119].

The electronic band of the MoO_3_ and crystal structure have an important effect on photocatalytic performance [19]. Thus, oxygen vacancies are thought to be an important contributor to this process’s effectiveness. These vacancies function as an electron trap, capturing the photogenerated electrons and suppressing the photoexcited carrier recombination [58,123,124]. “Vacancy doping” consists of the strategy of introducing free holes or free electrons for a higher charge carrier density without the need for extrinsic dopant ions [123]. MoO_3_ materials allow an adjustable oxygen vacancy density due to the special nature of their outer-d valence electrons, and these oxygen vacancies in MoO_3−x_ cause an extra defect band below the conduction band that decreases its effective bandgap as a consequence of the creation of the intermediate band (IB) in the gap [1,23,124]. The insertion of oxygen vacancies causes the creation of Z-type heterojunctions, and MoO_3−x_-based materials can generate high-energy electrons to participate in redox reactions [58]. Some synthetic methods for inserting oxygen vacancies include chemical reduction, ion intercalation, chemical vapor deposition, and ball milling treatment [1,124,125]. By modifying and altering the ball-milling periods, for instance, the oxygen vacancy content on the surface of α-MoO_3_ can be adjusted [125]. In the synthesis of MoO_3−x_, oleylamine is also reported to be utilized as a weak reducing agent, causing the formation of Mo^5+^ and consequent oxygen vacancies [19].

Different heterostructures can be synthesized with MoO_3−x_ for higher photocatalytic performances. For example, the matching band structure of sulfur-doped carbon nitride (S-CN) and MoO_3−x_ is expected to construct an S-scheme heterojunction catalyst. Because S and O atoms have similar electronegativity, during heterojunction creation, lone pair electrons of S atoms on S-CN can occupy the oxygen vacancy electron defect state on the MoO_3−x_ surface. By regulating the S-CN ratio, it is possible to maintain the proper oxygen vacancies while controlling the surface defect density of MoO_3−x_. This can help promote charge carrier separation as well as the adsorption and activation of molecular oxygen. The metal Mo atoms in MoO_3−x_ can interact with the lone pair electrons of the N atoms in S-CN, acting as electron donors, to form a covalent Mo(δ+)–N(δ-) bond with minimal space constraints. The charge transfer barrier can be lowered and the charge transfer rate accelerated by using this interfacial chemical bond as a particular “highway” [126].

In addition, researchers have been interested in improving the photocatalytic activity of polymeric carbon nitride (PCN) by coupling it with molybdenum-based compounds employed as co-catalysts [123]. Because g-C_3_N_4_ is positively charged and MoO_3_ is negatively charged, an electric field is formed between them. This built-in electric field, along with the bending direction of the band edge because of the nature of the electrons obtained, results in a Z-scheme carrier transfer mechanism. This indicates that the photogenerated electrons in the MoO_3_ conduction band can easily move and recombine with the holes in the g-C_3_N_4_ valence band under light irradiation, facilitating photogenerated carrier separation efficiency [112].

Embedded Mo reduces the PCN band gap and increases its valence band position, which improves visible light absorption and oxidation ability. When exposed to visible light irradiation, the Mo-embedded PCN creates photogenerated carriers, and the embedded Mo^6+^, as an electron transfer carrier, reduces the recombination. Three factors improve the photocatalytic activity considerably. First, the embedded Mo^6+^ reduces the recombination of photogenerated carriers by shortening the migration distance of electrons from the excited to the reactive sites. Second, the embedded Mo^6+^ lowers the PCN band gap, allowing it to capture more visible light irradiation. The valence band potential of PCN is increased, and the oxidizing ability of the photogenerated holes is improved. Third, more adsorptive and photocatalytic active sites emerge from the increased specific surface area. Because of the suitable and tunable band gaps between the semiconductors, their larger surface area, platinum-like electronic structure, and high electron storage capacity, semiconductors containing Mo, such as MoO_3_ and MoO_2_, can improve the interfacial transport of photogenerated carriers, resulting in improved photocatalytic activity. The chemistry related to the multivalent nature of Mo can explain the Mo effect. The electron mobility of the system is improved, and the internal resistance is reduced because of the Mo species with abundant d-electrons [127].

**Table 4:** MoO_3_ and MoO_3_-based systems used in photocatalysis applications.

Material	Morphology	Synthesis Method	Adsorbent Nature	Target	Lamp/Power	Time (min)	Removal Efficiency (%)	Reference
α-MoO_3_	Nanoparticles	Hydrothermal	Cationic dye	MB	LED (12 W)	90	99	[128]
α-MoO_3_	Plate-like	Pechini-based	Cationic dye	RhB	160 W	15	93.3	[129]
α-MoO_3_	Nanorods	Hydrothermal	Cationic dye	MB	Xenon (300 W)	120	98	[130]
α-MoO_3_	Nanorods	Hydrothermal	Cationic dye	RhB	Xenon (300 W)	120	90	[130]
α-MoO_3_	Microfibers	Hydrothermal	Cationic dye	MB	Halogen (150 W)	120	90	[131]
g-C_3_N_4_-tourmaline-α-MoO_3−x_	Nanosheets	Hydrothermal	Cationic dye	RhB	Xenon (300 W)	40	~100	[58]
g-C_3_N_4_-α-MoO_3_	Nanosheets/Nanoparticles	Mixing/Annealing	Cationic dye	RhB	Xenon (300 W)	24	100	[112]
α-MoO_3_-Cu_2_S	Nanoparticles/Hexagonal rods	Hydrothermal	Cationic dye	MB	Metal-halide (200 W)	120	98	[116]
α-MoO_3_-SiO_2_	Monoliths	Sol–Gel/Wet impregnation	Cationic dye	RhB	CFL/(45 W)	150	88.6	[118]
α-MoO_3_-NiO	Nanobelts/Nanoparticles	Hydrothermal	Cationic dye	MB	Xenon (500 W)	120	96.5	[120]
α-MoO_3_	Nanoparticles	Hydrothermal	Anionic dye	Eosin Yellow	LED (12 W)	90	94	[128]
α-MoO_3_	Nanorods	Hydrothermal	Anionic Dye	Alizarin	Xenon (300 W)	120	74	[130]
Ag-α-MoO_3_-TiO_2_	Spherical particles	Hydrothermal	Anionic dye	MO	UV light (100 W)	330	75.8	[132]
g-C_3_N_4_-α-MoO_3_	Nanosheets/Nanoparticles	Mixing/Annealing	Anionic dye	MO	Xenon (300 W)	40	82.3	[112]
α-MoO_3_-WO_3_	Nanorods	Hydrothermal	Metal ion	Cr^6+^	Tungsten (150 mW/cm^−2^)	25	97.6	[110]
α-MoO_3_@ZIF-8	Nanowires/nanoparticles	Hydrothermal	Metal ion	Cr^6+^	Sun 2000	40	100	[133]
MWCNT-α-MoO_3_	Nanotubes/Nanoparticles	Hydrothermal	Metal ion	Cr^6+^	Xenon (300 W)	30	100	[134]
α-MoO_3_-Fe_2_O_3_	Nanorods	Hydrothermal	Drug	Tetracycline	UD ^a^	90	96.5	[111]
α-MoO_3_-SiO_2_	Monoliths	Sol-Gel/Wet impregnation	Drug	Metronidazole	CFL/(45 W)	180	67.4	[118]
PPy-α-MoO_3_	Spherical like Particles/Platelike	In situ polymerization	Drug	Acetaminophen	Xenon (500 W)	180	93.4	[121]
g-C_3_N_4_-α-MoO_3_	Particles	Impregnation and calcination	Herbicide	2,4-dichlorophenol	Natural sunlight	300	99	[119]

^a^ Unavailable data.

**Figure 13:**
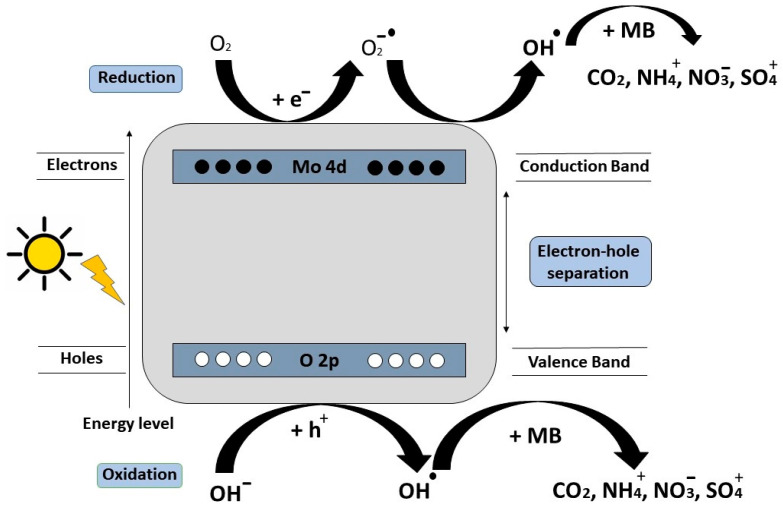
Schematic representation of visible light photocatalytic degradation of MB dye by MoO_3_.

Some organic dye photodegradation mechanisms over MoO_3_ materials can be proposed. For example, the MB photodegradation possibly occurs through α-MoO_3_ photoexcitation, resulting in an electron-hole pair generation on the surface [131]. Through the use of the corresponding trapping scavenges, it is indicated that hole radicals (h^+^) and hydroxyl radicals (OH^−^) are the primary reactive species acting as strong oxidants. On the other hand, according to the literature, the superoxide anion radicals ($O_{2}^{-.}$) have a minor role in this photodegradation process [116,131]. The redox reactions that take place in the visible-light photocatalytic mechanism for MB photodegradation are schematically represented in Figure 13. In the RhB photodegradation, the superoxide anion radicals ($O_{2}^{-.}$) and hole radicals (h^+^) play the main role as active species, with a small number of hydroxyl groups (OH^−^) also involved in the process reactions [111,130]. Thus, only some of the holes are used to oxidize water, while most of them are used for RhB photodegradation [58]. The phenomenon obeys an indirect mechanism, with strong oxidizing agents generated on the surface of the catalyst. These oxidizing agents react homogeneously with the organic material, resulting in its degradation [128]. During the Alizarin (AZ) photodegradation, the superoxide radicals ($O_{2}^{-.}$) act as active radical species, while the hole radicals (h^+^) do not act as active radicals [130].

## 5. Conclusions

This brief review comprised the latest advances in technological applications of MoO_3_ and MoO_3_-based materials in gas sensors, lithium-ion batteries, adsorption, and photocatalysis. Due to its electrochemical activity, high dielectric constant, great gas molecules adsorption, and charge carrier concentration modulation in response to gases, MoO_3_ is strongly indicated as a potential material as a gas sensor for NO_2_, NO, H_2_, CO, C_2_H_5_OH, NH_3_, trimethylamine, triethylamine, acetone, 1-butylamine, and water (humidity sensor), presenting a different sensing mechanism in comparison with typical n-type semiconductors. The reactions proposed in the MoO_3_ materials are a lattice oxygen reaction instead of a traditional surface-chemisorbed oxygen mechanism due to their crystalline-layered structure. However, the insufficient electrical conductivity of MoO_3_ restricts its electrochemical performance, stimulating investigations aimed at improving its performance with functional and surface modifications, morphological and sizing adjustments, the introduction of doping elements, the loading of noble metals, and the synthesis of composites. The MoO_3_ crystal is advantageous for lithium-ion intercalation because of the weak Van Der Waals force between the stacked sheets along the [0 1 0] direction. The intercalation ability and layered structure also provide diffusing channels and embedding sites for Li^+^ ions, making molybdenum oxides excellent options as cathode and anode materials for lithium-ion batteries, presenting higher theoretical specific capacities and faster discharge potential compared to conventional graphite. In this context, the weak electrical conductivity and slow diffusion kinetics of Li^+^ ions are often the limitations of MoO_3_. However, to overcome these problems, effective strategies, such as the diffusion barrier and band gap reduction, and the shortening of the electron and Li^+^ diffusion channels, have been researched. Molybdenum-based materials have also been reported for efficient photocatalytic applications, showing great promise in dye and antibiotic degradation under visible and ultraviolet irradiation. In particular, the oxygen vacancy creation induces an additional defect band that decreases its effective bandgap as a consequence of the intermediate band creation in the gap, acting as an electron trap and retarding the recombination. In particular, these materials have a strong oxidation capacity in the photocatalytic process due to their low valence band level. However, their rapid electron-hole recombination and limited absorption of visible light impose restrictions on their applications. To overcome these challenges and improve photocatalytic performance, doping of these semiconductors is a considered strategy since the formation of heterojunctions between different individual band gaps creates intermediate energy levels in the band gap region, facilitating the electronic transition and promoting efficient separation. of electron-hole pairs, in addition to delaying recombination. MoO_3_-based materials also present high adsorption performance and efficiency for pollutants removal through adsorption processes due to the abundant and fully exposed active sites on the oxygen-rich structure of MoO_3_, presenting high adsorptive capacity, especially towards cationic dyes because of its negatively charged surface, and heavy metal ions such as Pb^2+^, Cr^3+^, Cr^6+^, Cu^2+^, and Mn^2+^. On the other hand, for the adsorption of anionic dyes, research has revealed a viable option to produce composites by interspersing the layered structure of MoO_3_ with positively charged active sites. It is then possible to confirm the high applicability of MoO_3_-based materials in recent and important technological applications. We mainly outlined the applications of MoO_3_ in the fields of energy conversion devices, gas sensors, and catalysts. With regard to energy storage and conversion devices, more promising studies must be conducted since they have a bottleneck to ensure both capacitance and durability. As for gas sensors, though the MoO_3_ exhibits a good response to various gases, it is still necessary to investigate the sensing performance under different humid conditions to establish the relationship between the sensing properties and the environmental conditions. The catalytic nature and mechanism of MoO_3_-based catalysts are not fully understood, and accurate studies on the structure, phase, morphology, hybridization, and oxygen vacancies of MoO_3_-based materials must be conducted.

## Figures and Tables

**Table 3:** MoO_3_ and MoO_3_-based systems used in adsorption applications.

Adsorbent	Morphology	Synthesis Method	Adsorbent Nature	Target	q_m_ (mg·g^−1^)	Reference
α-MoO_3_	Nanobelts	Solvothermal	Cationic dye	MB	1408	[97]
α-MoO_3_	Nanobelts	Hydrothermal	Cationic dye	MB	UD ^a^	[100]
α-MoO_3_	Platelet-like crystals	Calcination	Cationic dye	RhB	26.56	[101]
α-MoO_3_	Platelet-like crystals	Calcination	Cationic dye	MB	152.7	[101]
α-MoO_3_	Platelet-like crystals	Calcination	Cationic dye	CV	199.4	[101]
α-MoO_3_	Platelet-like crystals	Calcination	Cationic dye	MG	53.05	[101]
α-MoO_3_	Micro/nanoplates	Hydrothermal	Cationic dye	RhB	UD ^a^	[103]
α-MoO_3_	Micro/nanoplates	Hydrothermal	Cationic dye	MB	UD ^a^	[103]
h-MoO_3_	Nanosheets	Hydrothermal	Cationic dye	RhB	1242	[99]
h-MoO_3_	Nanosheets	Hydrothermal	Cationic dye	MB	1433	[99]
α-MoO_3_/h-MoO_3_	Nanoparticles	Internal combustion	Cationic dye	MB	141.2	[98]
α-MoO_3_-MoO_2_	Nanoparticles	Hydrothermal	Cationic dye	MB	1250	[22]
α-MoO_3_-MoS_2_	Porous core-shell nanorods	Hydrothermal	Cationic dye	RhB	326.83	[96]
α-MoO_3_-TiO_2_	Nanoparticles	Hydrothermal	Cationic dye	RhB	169	[102]
α-MoO_3_-TiO_2_	Nanoparticles	Hydrothermal	Cationic dye	MB	180	[102]
α-MoO_3_	Micro/nanoplates	Hydrothermal	Anionic dye	Eosin yellow	UD ^a^	[103]
Al_13_-MoO_3_	Flake-like	Polycationic Encapsulation	Anionic dye	MO	357.2	[107]
α-MoO_3_	Porous nanosheet array	Hydrothermal	Metal ion	Pb^2+^	1450	[94]
α-MoO_3_	Nanobelts	Solvothermal	Metal ion	Pb^2+^	684.93	[97]
SiO_2_-α-MoO_3_	UD ^a^	Thermal decomposition	Metal ion	Pb^2+^	222.2	[95]
α-MoO_3_-TiO_2_	Nanoparticles	Hydrothermal	Metal ion	Cr^3+^	59	[102]
Chitin-MoO_3_-Montmorillonite	Nanorods	Blending	Metal ion	Cu^2+^	19.03	[104]
Chitin-MoO_3_-Montmorillonite	Nanorods	Blending	Metal ion	Pb^2+^	15.92	[104]
CeO_2_–MoO_3_–SiO_2_(CH_2_)_3_-(Alginate)_2_	Nanoparticle	Combustion/microwave irradiation	Metal ion	Mn^2+^	122.06	[105]
CeO_2_–MoO_3_–SiO_2_(CH_2_)_3_-(Alginate)_2_	Nanoparticle	Combustion/microwave irradiation	Metal ion	Cr^6+^	151.96	[105]
MoO_3_/γ-Al_2_O_3_	Nanoparticles/Nanorods	Cation-anion double hydrolysis/Impregnation	Aromatic sulfur compound	Dibenzothiophene	UD ^a^	[106]
MoO_3_/γ-Al_2_O_3_	Nanoparticles/Nanorods	Cation-anion double hydrolysis/Impregnation	Aromatic sulfur compound	Thiophene	UD ^a^	[106]

^a^ Unavailable data.

## Data Availability

Not applicable.
